# Case report: Blood purification effectively relieves multiple system failure in patient with rabies

**DOI:** 10.3389/fpubh.2022.979828

**Published:** 2022-10-26

**Authors:** Daibin Kuang, Ming Luo, Jiabao Chen, Congmin Liang, Ziwei Cai, Zeqiang Yuan, Zhuandi Zhou, Jialong Chen, Chunlai Fu

**Affiliations:** ^1^Department of Emergency Intensive Care Unit, Affiliated Dongguan People's Hospital, Southern Medical University, Dongguan, China; ^2^Department of Environmental and Occupational Health, School of Public Health, Guangdong Medical University, Dongguan, China

**Keywords:** case report, blood purification, multiple system failure, rabies, antiviral treatment

## Abstract

Rabies is an infectious disease of animal origin with a high mortality rate. In the early stages of rabies, the rabies virus (RABV) is usually undetectable in saliva and cerebrospinal fluid (CSF). In addition, there are still no effective drugs and treatments. Here, we present a case in which blood purification alleviated multisystem failures. The patient was a 45-year-old woman who presented with the fear of water and wind, restlessness, and hyperactivity. RABV was detected in her saliva by high-throughput sequencing Next Generation Sequencing (NGS) and polymerase chain reaction (PCR). Based on typical clinical symptoms and the result of NGS and PCR, the patient was diagnosed as a confirmed case of rabies. Hemodialysis combined with antiviral therapy and intensive care unit (ICU) treatment can effectively relieve circulatory failure, respiratory failure, and renal failure. Finally, she died of brain death on the 34th day of admission. The case report showed that blood purification was positive for rabies-induced organ failure. Blood purification combined with antiviral therapy can prolong the lives of patients with rabies to some extent.

## Introduction

Rabies is an infectious disease of animal origin caused by rabies virus (RABV), usually resulting in viral encephalitis. With an almost 100% mortality rate, rabies is the deadliest infectious disease in the world. Currently, there are still no effective drugs and treatments for rabies. WHO considers proper pre-exposure prophylaxis (PREP) and post-exposure prophylaxis (PEP) to be essential to prevent rabies deaths in humans and recommends increasing vaccination rates of dogs to 70% to prevent the spread of the rabies vaccine virus (1). However, the national monitoring data in China from 2012 to 2018 showed that the immunization coverage rate for dogs was only 40%, especially since lots of stray dogs are not vaccinated, which cannot effectively suppress the spread of rabies (2–4). In a case analysis of rabies, the post-exposure vaccination rate of patients was only 8.13% (5). In addition, the difference in the location, the degree of rabies exposure, and the timeliness of post-exposure treatment can affect the effectiveness of vaccination, and even rabies may occur after PEP treatment. Therefore, finding a reasonable and effective rabies treatment is a key challenge and an urgent task for medical research. In this study, we found that blood purification was effective in relieving multisystem failure in patients with rabies. The following clinical data are discussed and analyzed.

## Case information

### Preadmission information

A 45-year-old woman was hospitalized on June 8. Her husband reported that she had non-projectile vomiting and wheezing the previous day. Even the symptomatic treatment given by the doctor did not ease her symptoms. She feared wind and water and felt restlessness and hyperactivity, without headache, dizziness, palpitations, and coma. She was admitted to the emergency intensive care unit (ICU) on June 9. Routine physical examination and infection index (erythrocyte sedimentation rate, procalcitonin (PCT), and C-reactive protein), organ function, and arterial blood gas analysis were performed before admission. The results showed that the vital signs were all within the normal range, with clear thinking, hyperactive irritability, and fear of wind and water. The following indicators are abnormal in the auxiliary test: PCO2: 16 mmHg; BE: −9.3 mmol/L; and Lac: 7.2 mmol/L. All other indicators are normal. The patient told us that she had a cat 2 years ago with no history of animal bites or scratches.

### Post-admission data and disease progression

Before admission to the hospital, she had fear of wind and water, restlessness, and hyperactivity. Other common infections (bacteria, fungi, and parasites) were tested and showed negative. The multidisciplinary consultation agreed that there is a great possibility for rabies.

Preliminary therapeutic ideas: doctors informed the family that the patient was very ill. A single room for isolation prevented in-hospital infection. Sedation, assisted respiration by endotracheal intubation, stomach protection, liver protection, and nutritional support were used in the patient. Monitoring blood gas analysis, blood biochemistry, infection indicators, and organ functions are implemented. Patients were treated with antiviral therapy to look for evidence of etiology.

Antiviral therapy: On day 1 (6.09), the patient was treated with Ribavirin (1 g, *via* intravenous injection); from day 2 (6.10) to day 10 (6.18), the patient was treated with ribavirin (0.5 g, *via* intravenous injection).

Measures to mitigate adverse reactions in the patient included: (a) endotracheal intubation ventilator assists breathing and protects the airway; (b) strengthen sedation and analgesia to control convulsions; and (c) blood purification (using Diacap Acute M as hemofiltration and CVVH as the filter type).

Measures on anti-shock included: (a) early fluid replacement to correct hypovolemia; (b) use of vasoactive drugs such as norepinephrine to constrict blood vessels; and (c) use of low-dose ulinastatin to suppress inflammatory responses.

Disease progression:

On day 1 (6.09), the patient presented with a high fever, with a temperature of up to 40°C, accompanied by convulsions and hypotension. High-throughput sequencing of the blood and cerebrospinal fluid (CSF) showed no abnormalities. Patients were given intensive analgesia, sedation, fluid replacement, vasoactive drugs, prophylactic anti-infectives, hormone therapy, anti-viral agents, hemoperfusion, and hemofiltration. In addition, a lumbar puncture was performed to rule out intracranial infection.

On day 2 (6.10), she still has intermittent convulsions and increased doses of painkillers, sedatives, and muscle relaxants. The doctor performed an auxiliary examination on her and found many abnormal indexes.

The biochemical examination of the spinal cord revealed increased glucose (GLU) concentration (7.46 mmol/L) and low CSF lactate dehydrogenase (LDH; 18.5 U/L).

Routine C-reactive protein (RT-CRP; 36.87 mg/L), white blood cell count (18.51 × 10^∧^9/L), neutrophil percentage (84.7%), and B-type natriuretic peptide (BNP; 3,456 pg/ml) were elevated.

Myocardial marker tests revealed elevated high-sensitivity troponin (33,855.2 pg/ml), myoglobin (2,483.4 ng/ml), and PCT (11.55 ng/ml).

Activated partial thromboplastin time (57.2 s) and thrombin time (70.0 s) were prolonged; D-D dimer (10.10 μg/ml) and fibrinogen degradation products (31.18 μg/ml) were increased.

The value of GLU (G32LU) was 9.58 mmol/L, which is high.

The results indicated as follows: aspartate aminotransferase (AST) 234 U/L, LDH 659 U/L, phosphocreatine kinase (CK) 8,419 U/L, phosphocreatine kinase isoenzyme (CKMB) 300.0 U/L, lipase (LIPA) 422 U/L, and amylase (AMYL) 959 U/L.

The thyroid function test showed that thyrotropin (TSH) was 0.22 mIU/L, which is low.

Liver function test results indicated as follows: alanine aminotransferase (ALT) 103.1 U/L and AST 279.9 U/L.

Toxic cardiomyopathy was considered based on the above clinical and laboratory findings. The possibility of rabies remains to be diagnosed. The exact cause remains to be determined, and high-throughput sequencing of the CSF continues to be pursued.

On day 5(6.13), she still has a high fever, with a body temperature of up to 40°C; there was still no improvement in cardiac function index and infection index; white blood cell (WBC) was still continued to rise based on CRP and PCT. Her medication was adjusted, and she was given “Meropenem + Kauranen” for anti-infection treatment. She was given treatment with sedation and analgesia, ventilator-assisted respiration, anti-infectives, antiviral agents, diuretic dehydration, anti-shock therapy, liver and stomach protection, nutritional support, and antipyretic drugs.

On day 6 (6.14), her cardiac color ultrasonography suggested an ejection fraction of 18%. We treated her with levosimendan, noradrenalin + dobutamine, controlled rehydration, and hemofiltration therapy intermittently.

On day 7 (6.15), the patient developed intermittent fever with a temperature as high as 39.5°C. The indicators were reviewed and the following abnormalities were observed:

BNP >5,053 pg/ml; high-sensitivity troponin I (hsTnI) 1,914.6 pg/ml and myoglobin (Myo) 2,492.3 ng/ml; RT-CRP 131.14 mg/L, leukocytes 21.78 × 10^∧^9/L, neutrophil 88.80%, lymphocyte 4.70%, hemoglobin 110.00 g/L, and PLT 94.00 × 10^∧^9/L; PCT 10.11 ng/ml; D-dimer > 20 μg/ml and fibrin (proto) degradation product (FDP) 68.10 μg/ml.

Blood electrolyte: Ca^+^ 2.06 mmol/L.

Renal function test results: urea 21.84 mmol/L, creatinine (CR) 154.7 μmol/L, and GLU 13.65 mmol/L.

Cardiac enzymes: AST 89.2 U/L, LDH 575.5 U/L, creatine phosphokinase (CK) 3,000.6 U/L, and CK-MB 54.5 U/L.

Liver function tests: ALT 121.8 U/L, AST 88.5 U/L, γ-glutamyl transpeptidase (γ-GGT) 309.3 U/L, alkaline phosphatase (ALP) 145.8 U/L, LDH 553.9 U/L, and albumin (ALB) 35.1 G/L.

We took into account the current clinical presentation of the patient, who is in critical condition. The patient is given hemofiltration therapy (cleared the inflammatory mediators) and the rest of the treatment is the same as before.

**Figure 1:**
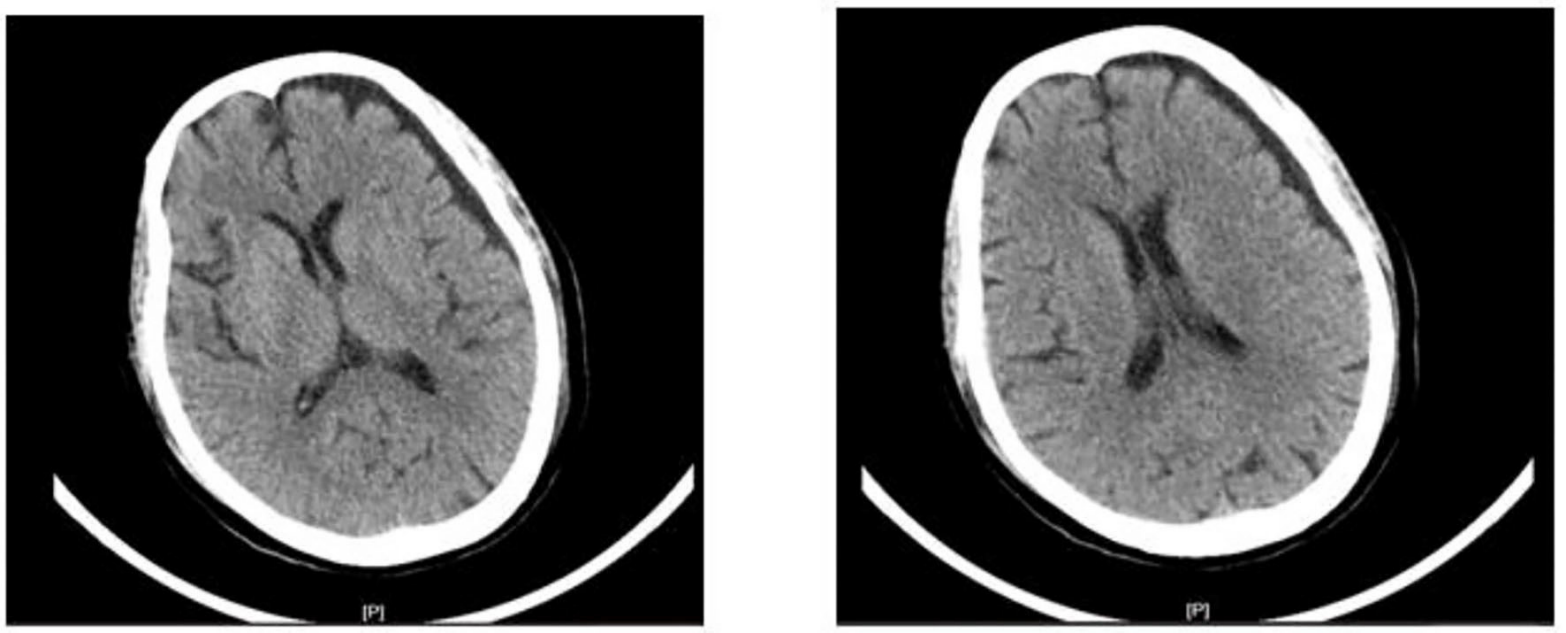
The computed tomography image of the brain on day 12.

On day 12 (6.19), the patient had no further convulsions and went into a deep coma. There were a lot of salivary secretions, abdominal distention, and bowel irregularities. Therefore, her treatment measures were adjusted and analgesic sedatives and inotropic drugs were stopped. At the same time, the results of the CT suggest that the patient has brain atrophy (Figure 1).

On day 17 (6.24), a high-throughput sequencing test of the patient's saliva resulted in the detection of RABV (Table 1). Thus, the patient was diagnosed with rabies.

**Table 1:** The result of a high-throughput sequencing test of the patient's saliva.

**Type**	**Species**	
	**Name**	**Detected sequence**
ssRNA	Rabies lyssavirus	3,075

On day 20 (6.27), multiple sputum cultures indicated pan-drug-resistant *Acinetobacter baumanii*.

On day 22 (6.29), urine output gradually decreased to <400 ml/day and diuretic use was ineffective. We can control the fluid infusion, use a natriuretic peptide, and continue the hemofiltration-based treatment.

On day 24 (7.01), the heart rate slows down to a maximum of only 35 beats/min.

On day 34 (7.11), Glasgow Coma Scale score was found to be 3, no spontaneous breathing was observed, and high-dose vasoactive drugs were used to maintain blood pressure. Her heart rate was 60 beats/min. She has multiple organ failures. On the next morning, the patient's heart rate slowed to 20 beats/min followed by a decrease to a heart rate of 0. Electrocardiogram (EKG) shows that blood pressure was 0 mmHg and blood oxygen degree of fingertips was 0%. On the physical examination, the patient was in deep coma with bilaterally dilated pupils, with 6.0 mm in diameter. The patient's light reflex and carotid pulse are both absent. We cannot hear the patient's cardiechema signals. She did not recover for 30 min and was pronounced clinically dead. Important findings developed as follows (Table 3).

In the early stages, combining with clinical presentation (preadmission) and the saliva result of NGS (6.24), the patient was diagnosed with rabies. On day 25 (7.02), saliva and CSF were tested by PCR (Table 2), and the sample of saliva showed positive for rabies lyssavirus. In PCR, a pair of primers were designed for the most conserved region of the RABV nucleoprotein (NP) gene: N1(+): (587)5′-TTT GAG ACT GCT CCT TTT G (605)-3′; N2(-): (1092)5′-CC CAT ATA GCA TCC TAC (1013)-3′. The result of PCR can be exactly confirmed as the result of NGS and diagnosed the patient with rabies.

**Table 2:** The result of polymerase chain reaction of rabies lyssavirus.

**Sample**	**Nucleic acid test results**
Saliva	Positive
Cerebrospinal fluid	Negative

**Table 3:** The change of patient indicators during observation.

**Date**	**Maximum temperature (°C)**	**Urine volume (mL)**	**Hs-cTn (ng/L)**	**White blood cell (10^∧^9/L)**	**Infection index**		**Myocardial enzyme (U/L)**		
					**CRP (mg/L)**	**PCT (ng/mL)**	**CK**	**CK-MB**	**Mb**
6.09	38	3,445	11,200	28.47	22.7	3.71	5,606	135	4,144
6.10			46,025	18.51	36	11.5	10,024	268	2,483
6.11	39	2,755	54,865	13.77	133	5.37	3,681	67	322
6.12			20,318	13.36	145	7.46	2,529	63	685
6.13	39.5	2,300	5,225	19.57	128	10.11	2,343	40	1,085
6.14			3,649	19.74	108	7.24	1,585	45	1,398
6.15	37.6	2,137	1,914	21.74	123	4.59	3,000	54	2,492
6.16			1,347	21.25	89	2.62	3,112	57	1,730
6.17	38	3,745	1,424	18.83	101	1.69	4,215	36	1,988
6.18			4,125	19.61	137	3.51	4,213	38	4,144
6.19	37.8	1,000	2,292	23.89	136	3.61	4,318	46	4,144
6.20			1,754	21.44	132	7.22	4,897	70	4,144
6.21	36.5	1,530	1,457	18.9	137	6.90	4,952	78	4,144
6.22			1,519	14.53	165	2.43	6,592	60	4,144
6.23	39	1,780	3,271	19.99	128	2.98	6,725	61	4,144
6.25	38	1,930	2,280	17.03	135	2.98	10,268	92	4,144
6.27	37.6	1,535	4,423	13.33	136	3.02	8,625	79	4,144
6.29	37	275	4,567	8.92	178	6.33	5,361	137	4,144
7.1	39.5	185	3,246	9.36	136	9.91	3,273	210	4,144
7.3	38	125	1,543	6.94	200	6.78	3,526	165	4,144
7.5	36.9	30	2,449	4.61	129	7.23	3,220	108	4,144
7.7	37	45	2,986	9.64	104	10.21	2,194	70	4,144
7.9	36.8	56	2,520	8.64	108	15.23	1,018	76	4,144
7.11	36.5	46	2,729	6.25	97	14.23	864	56	4,144

## Discussion and conclusion

### Discussion

The incubation period of rabies is usually 2–3 months or may range from <7 days to 6 years (6, 7). Patients with head and neck bites have a shorter incubation period, while bite sites have a longer incubation period in the limbs (7). This case may be a patient with a long incubation period of manic rabies, and the incubation period is 1–2 years. At the time of admission, she would show panic if she encountered wind or had contact with water. High-throughput sequencing (NGS) of saliva and CSF was performed immediately after admission and was negative for the first time. On the 15th day of hospitalization, the saliva was positive for NGS, and the rabies sequence was 3,075. Meanwhile, PCR also verified the positive result of the virus. In the early stages of rabies, the virus cannot be detected in saliva and CSF. Therefore, a definitive diagnosis can be difficult. With high sensitivity and specificity, NGS can detect 10's of 1,000's of pathogens with one detection (8). The patient in this case was diagnosed with rabies by combining with clinical presentation and the result of NGS and was finally exactly diagnosed with rabies by PCR.

The mechanism by which RABV damages the central nervous system remains unclear. In the early days, the Milwaukee plan was widely touted. Between 2005 and 2014, all 29 human rabies cases recorded in the United States, Canada, and the United Kingdom were treated with the Milwaukee protocol (9, 10). Only two patients treated during this period have been reported as survivors. With such a low success rate, the treatment has been abandoned by many doctors. WHO also does not recommend coma-inducing therapy (11). There is also evidence that the therapy fails to clear rabies from the brain. After the onset of rabies, the patient usually dies within a week, rarely more than 10 days (12). The current research progress on rabies treatment shows that RABV antibodies can efficiently neutralize RABV. However, RABV antibodies cannot neutralize RABV in the central nervous system causing the blood–brain barrier (13). Most of these cases die of organ failure. The key to rescue the patient with rabies is supposed to clear the virus in the central nervous system (14). However, once the disease has developed, doctors can only treat the symptoms and relieve the patient's suffering. Studies have shown that strong intensive care teams can prolong the lives of patients with rabies. We have effectively alleviated circulatory failure, respiratory failure, and renal failure through hemodialysis combined with antiviral therapy and ICU. The patient finally died of brain death on the 34th day of admission. Blood purification can remove the free light chain (FLC) from the circulatory system, which may reduce kidney damage and promote recovery of kidney function (15). At the same time, some studies have pointed out that continuous blood purification can promote the body's electrolyte balance and acid-base balance, so that patients with azotemia and fluid balance can be controlled (15, 16). Studies also found that blood purification can promote the stability of the internal environment, reducing the peak concentration of inflammatory cytokines (15, 17).

### Conclusion

Antibodies or viruses may not be detectable in the early stages of rabies and should be tested multiple times in a row. Blood purification is positive for organ failure caused by rabies. The use of blood purification combined with antiviral treatment for patients with rabies, under the management of the intensive care team, can greatly extend the life of patients with rabies, for the treatment of rabies to win precious exploration time.

## Data availability statement

The original contributions presented in the study are included in the article/supplementary material, further inquiries can be directed to the corresponding authors.

## Ethics statement

The studies involving human participants were reviewed and approved by Dongguan People's Hospital, Southern Medical University, Dongguan, Guangdong, China. The patients/participants provided their written informed consent to participate in this study. Written informed consent was obtained from the individual(s) for the publication of any potentially identifiable images or data included in this article.

## Author contributions

JialC and CF contributed to conception and design of the study. CF, DK, ML, JiabC, ZY, and ZZ organized the therapy and experiment. JialC wrote the first draft of the manuscript. CL and ZC wrote sections of the manuscript. All authors contributed to manuscript revision, read, and approved the submitted version.

## Funding

This work was supported by the National Natural Science Foundation of China (82103879 and 81502899), the Guangdong Basic and Applied Basic Research Foundation (2021B1515140032), the Discipline Construction Project of Guangdong Medical University (4SG21021G and 4SG22001G), and the Science Foundation of Traditional Chinese (20222104).

## Conflict of interest

The authors declare that the research was conducted in the absence of any commercial or financial relationships that could be construed as a potential conflict of interest.

## Publisher's note

All claims expressed in this article are solely those of the authors and do not necessarily represent those of their affiliated organizations, or those of the publisher, the editors and the reviewers. Any product that may be evaluated in this article, or claim that may be made by its manufacturer, is not guaranteed or endorsed by the publisher.
